# Case Report: Therapeutic and immunomodulatory effects of plasmapheresis in long-haul COVID

**DOI:** 10.12688/f1000research.74534.2

**Published:** 2022-04-06

**Authors:** Dobri D. Kiprov, Ahvie Herskowitz, Daehwan Kim, Michael Lieb, Chao Liu, Etsuko Watanabe, Jan C. Hoffman, Regina Rohe, Michael J. Conboy, Irina M. Conboy

**Affiliations:** 1 California Medical Pacific Center, San Francisco, CA, 94109, USA; 2 Bioengineering, UC Berkeley, Berkeley, CA, 94720, USA; 3 Department of Laboratory Medicine, UCSF, San Francisco, CA, San Francisco, USA

**Keywords:** Immunomodulation, Long Haul Covid19, plasmapheresis, adaptive immunity, inflammation, proteomics, leukocyute subsets

## Abstract

Many patients with COVID-19 experience a range of debilitating symptoms months after being infected, a syndrome termed long-haul COVID. A 68-year-old male presented with lung opacity, fatigue, physical and cognitive weaknesses, loss of smell and lymphocytopenia. After rounds of therapeutic plasma exchange (TPE), the patient returned to normal activities and work. Mechanistically in the patient’s peripheral blood mononuclear cells (PBMCs), markers of inflammatory macrophages diminished and markers of lymphocytes, including natural killer (NK) cells and cytotoxic CD8 T-cells, increased. Circulating inflammatory proteins diminished, while positive regulators of tissue repair increased. This case study suggests that TPE has the capacity to treat long-haul COVID.

## Introduction

The symptoms of “long-haul” coronavirus disease 2019 (long COVID) are debilitating and prevent patients from working, which is projected to negatively impact the healthcare system and economic recovery. ^1^ The most common symptoms of long COVID are dyspnea with abnormal chest radiograph (CXR) findings, extreme fatigue, cognitive impairment (described as “brain fog”), myalgias, anosmia, ageusia, headache and sleep disorder. ^2^ Long COVID resembles myalgic encephalomyelitis/chronic fatigue syndrome (ME/CFS) which is driven by autoantibodies. ^3^^,^^4^ Therapeutic plasma exchange (TPE) was successfully tried in patients with severe COVID-19 through various methods ^5^^,^^6^ and is typically used on patients with ME/CSF, thrombotic thrombocytopenia, ^7^ and other autoimmune disorders. ^8^^,^^9^ Moreover, our recently published studies suggest that TPE re-sets the circulatory proteome to healthier states, attenuating the so-called cytokine storm and enhancing the systemic determinants of tissue repair. ^10^^,^^11^ The encouraging results of these reports convinced us to try TPE on a patient with severe long COVID.

## Case

A 68-year-old caucasian male attorney, who reported having been very physically active and capable of multitasking in a demanding executive position at work began to feel fatigued and short of breath on December 14, 2020. He visited an emergency room on December 18, 2020. His oxygen saturation was 90–93% on room air and he was sent home. His breathing and fatigue deteriorated, so he was admitted to the hospital on December 21, 2020. He tested positive for severe acute respiratory syndrome coronavirus 2 (SARS-CoV-2) by PCR and was treated with remdesivir and Decadron (dexamethasone). His status continued to worsen, and he was placed on high-flow oxygen. He was never intubated. After 11 days in the hospital, he was sent home on portable oxygen which he used intermittently.

**Figure 1.**
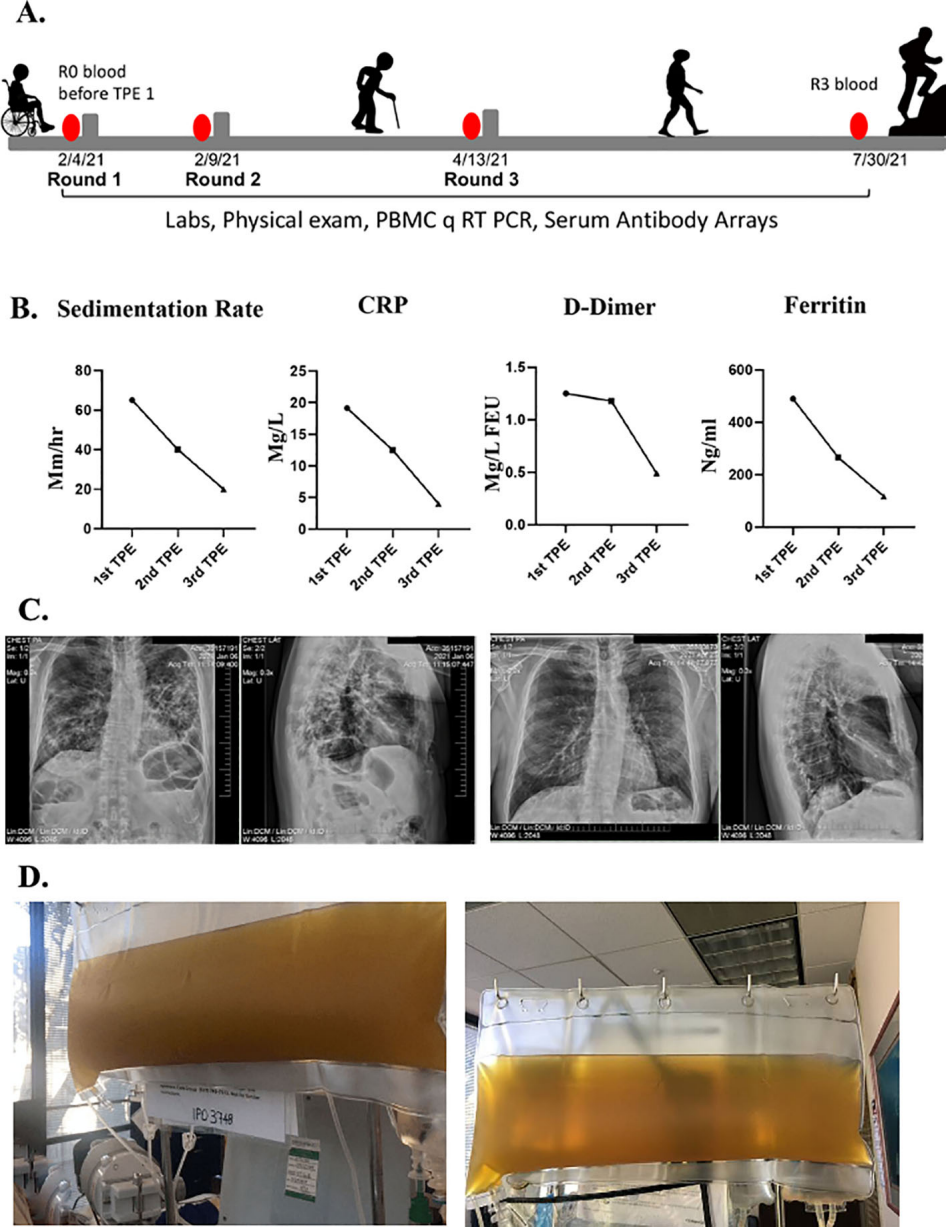
Clinical improvements in a long COVID patient. A. Schematics of the case. B. Erythrocyte sedimentation rate, CRP, D-dimer, ferritin levels were assayed before each TPE procedure and were initially elevated but normalized by the TPE. C. Chest radiographs show reduced lung opacity after TPE. D. Clouded plasma appearance was reduced by TPE.

Over the following four weeks, he felt extremely fatigued. He could only walk to the bathroom and back to bed. He could not focus on anything cognitively, which he described as “brain fog”. He was unable to do any work and could not even answer his emails. He reported he had no sense of smell nor taste. Sedimentation rate, CRP, D-dimer and ferritin were abnormal in clinical lab tests **(** Figure 1B). At that time, chest radiographs and a chest CT scan revealed ground-glass appearance areas diffusely spread throughout his lungs ( Figure 1C). A PCR test for SARS-CoV-2 was negative. A serologic test for anti-SARS-COV-2 IgG antibodies was positive.

In February of 2021, he was seen in our clinic. At that time, he was very weak and unable to walk. When he arrived at the airport, he needed a wheelchair to go from the plane to the taxi. He underwent his first TPE on February 4, 2021. One plasma volume was exchanged, using 5% albumin as an exchange fluid. The removed plasma was very dark and opaque ( Figure 1D). During his first TPE treatment, he was coughing profusely. After his first treatment, he could breathe more easily, and the cough subsided. The morning after the first TPE treatment, he could walk and was not struggling for breath. Two days later, he had no difficulty breathing and was able to walk 100 feet on a level surface but still had difficulty walking uphill.

He underwent a second TPE and two days after the second treatment he was able to walk uphill with ease and even jog. His brain fog disappeared, and he was able to get back to his daily work activities. Typical biomarkers of systemic inflammation all became quickly and robustly normalized including erythrocyte sedimentation rate, CRP, ferritin, and D-dimer ^12^ ( Figure 1B). The plasma from the second TPE was clear ( Figure 1D). A week later his chest radiographs showed considerable clearing of the opacities in the lungs ( Figure 1C). He also reported regaining his sense of smell and taste. He was seen in the clinic for another TPE two months later and, at that time, the patient reported that he was back to work, feeling like his normal self, and able to exercise daily without shortness of breath.

**Figure 2.**
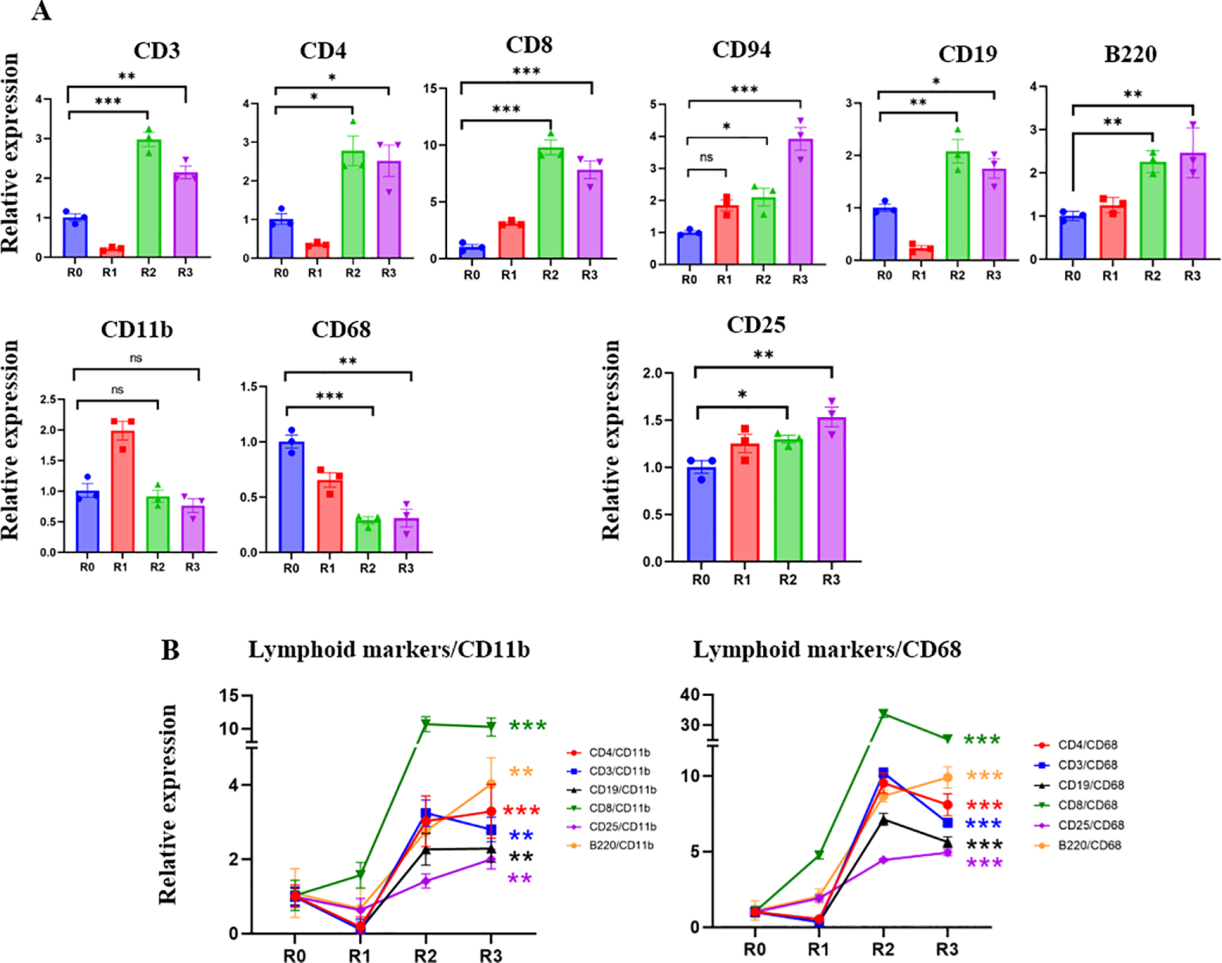
Effect of TPE on lymphoid and myeloid gene expression. A. The expression of T cell, B-cell NK cell markers and IL2 receptor are significantly increased in R2 and R3 compared to R0. CD11b myeloid marker increased in R1 and then returned to R0 levels. CD68 – the marker of inflammatory myeloid cells, was significantly decreased by the third round of TPE. B. The ratios of lymphoid/CD11b, lymphoid/CD68, CD25/CD11b and CD25/CD68 markers clearly demonstrate the positive effects of TPE in promoting the adaptive rather than the inflammatory immune response. Note the break in Y-axis scale. * *P* < 0.05, ** *P* < 0.01, *** *P* < 0.001.

The peripheral blood mononuclear cells (PBMC) and blood serum of this patient were collected and analyzed before the first round of TPE (R0) and before each subsequent round (R1–R3) **(** Figure 1A). Real-time qRT PCR was used to study the levels of the markers of T cells: CD3; helper T-cells: CD4; cytotoxic T-cells: CD8; NK cells: CD94; B-cells: B220 and CD19; macrophages: CD11b; inflammatory myeloid cells: CD68; and IL-2 receptor: CD25. Before TPE, there was a prevalence of CD68+ inflammatory myeloid cells’ marker, and relatively fewer lymphocyte markers, which is indicative of a lack of adaptive immunity ( Figure 2). TPE increased the markers of T-cells, B-cells, NK cells, and diminished the markers of inflammatory macrophages, suggesting enhanced adaptive immunity and attenuated inflammatory response ( Figure 2 **)**. CD3, CD4 and CD19 initially diminished at 5 days after the first TPE, R1, but then steadily increased during the longer intervals of R2 and R3; CD94 and CD8, the markers of cytotoxic immune cells that combat viral infections also gradually and steadily increased ( Figure 2).

Five days after the first TPE and the initial increase of CD11b+, the levels of this marker stabilized, while the CD68 marker of inflammatory macrophages were never elevated by TPE and were diminished by R2 and R3 ( Figure 2). Plotting the ratios of T-cell and B-cell markers to myeloid and inflammatory macrophage markers (CD11b and CD68) demonstrates a rapid and robust shift toward adaptive immunity through the rounds of TPE ( Figure 2 **)**. The effects of TPE are particularly striking when looking at the relative increase in the CD8+/CD68+, *e.g.*, anti-viral cytotoxic T cells in an inflammation reduced environment ( Figure 2 **)**.

**Figure 3.**
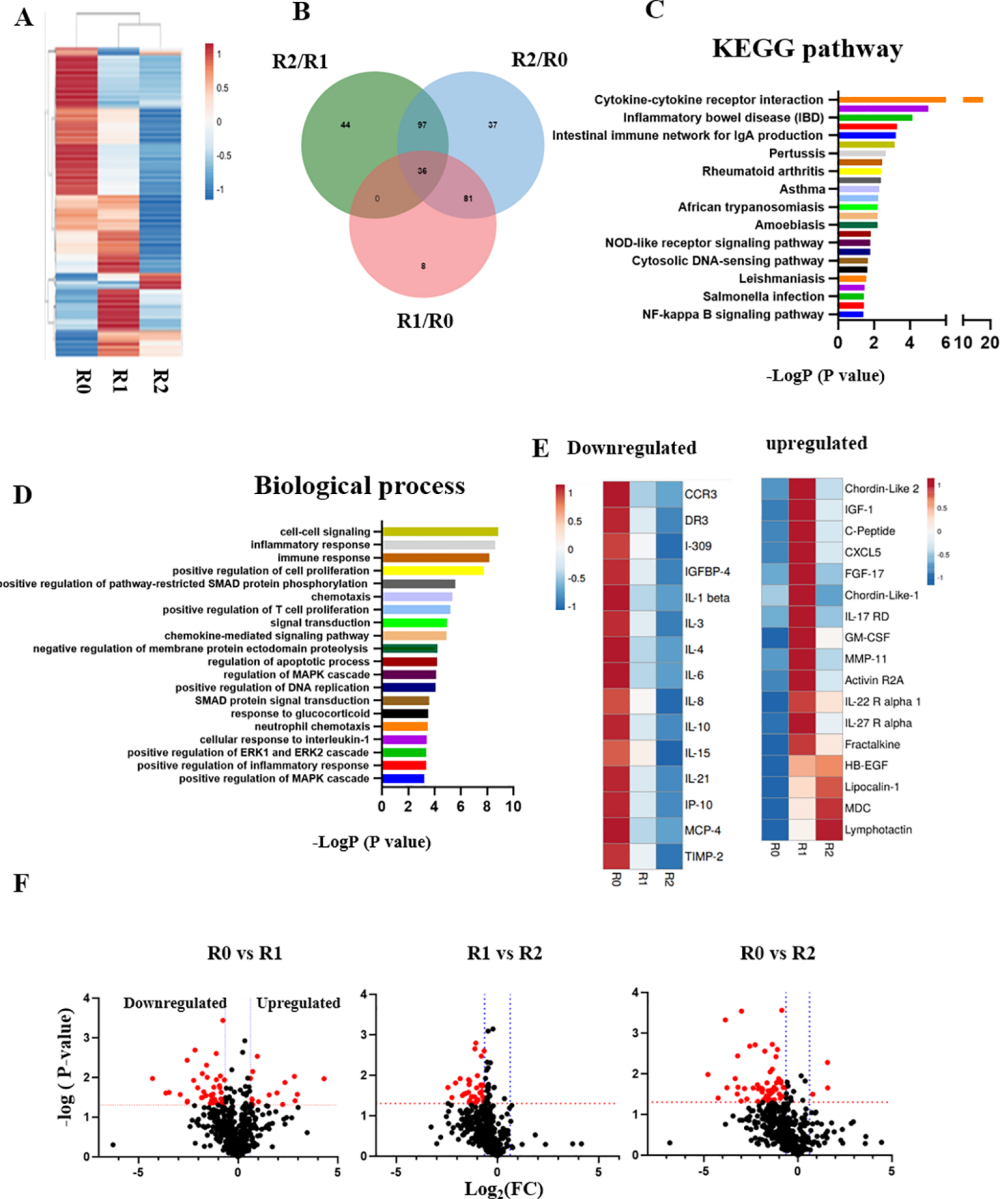
The longitudinal profiling of serum proteome after rounds of TPE. A. Comparative heat mapping of 507 proteins between TPE rounds was done on RayBiotech Array. B. Venn diagram showing the overlap between the proteins, which were influenced by the TPE rounds. 36 shared proteins were diminished each TPE round (0.65> Fold change). C. The KEGG signaling pathway analysis. D. The top 20 list of biological processes. E. Heat mapping of the proteins that relate to Aging and Inflammaging and are decreased by TPE. F. Volcano plots of proteomics as per TPE rounds. More proteins are downregulated by the second round than by the first. The red dots represent differently expressed proteins ( *P* < 0.05; |Log _2_FC|<1.5), while the grey dots represent proteins with *P* > 0.05.

Blood serum from the patient was profiled through comparative proteomics using RayBiotech antibody arrays, as we have published. ^10^ Heatmapping clearly demonstrated the profound influence of TPE on the circulatory proteome ( Figure 3A); Venn diagrams, KEGG and biological process databases uncovered 36 proteins that were commonly downregulated between the rounds of TPE, ( Figure 3B–D). In agreement with the diminished inflammation and enhanced adaptive immunity that were suggested by Figures 1 and 2, this longitudinal serum proteomics revealed significant attenuation of inflammatory factors, which was stable and lasted several months ( Figure 3E and F). Regulators of several growth factor pathways that are known to promote tissue repair increased in the systemic milieu: IGF, EGF, TGF-β ( Figure 3E and F).

These results establish rapid and significant changes in circulatory proteome of the patient, which are indicative of attenuated inflammation, productive immune response, and enhanced tissue maintenance.

Since the COVID-19 pandemic, roughly 70–80% of patients suffer various sequelae. ^13^ About 40% of these patients have acute respiratory distress syndrome (ARDS) and one of the main sequelae is pulmonary fibrosis. ^14^ Although the mechanism of COVID-19-induced ARDS may be different from classical ARDS, the onset and development of pulmonary fibrosis are commonly related to elevated inflammatory cytokines, such as IL-6. ^15^ Accordingly, anti-inflammatory drugs attenuate COVID-19-induced pneumonia. ^16^^,^^17^

In this study, a patient who was initially healthy, quickly developed health problems after being infected with the novel coronavirus. Moreover, after two months, his health deteriorated to the point of being unable to walk, being physically short of breath, suffering mental fatigue, and diffuse ground-glass appearance was observed throughout his lungs. Clinical and biological analyses strongly suggested systemic inflammation and a lack of productive immune response. After the first TPE treatment, the dyspnea disappeared, and cough subsided. The patient was also able to walk short distances. Interestingly, these changes were observed on day 2 after the first TPE treatment. A month later, the patient was able to walk uphill easily and jog. The decrease in concentration and cognitive fog disappeared, and the patient was able to return to normal life. During the same period, inflammatory macrophage markers robustly diminished and the markers of lymphocytes increased in the blood, suggesting that TPE promoted adaptive immunity and decreased inflammatory response. Such a productive immune response shift became prominent between the second and third rounds of TPE and rounds 1 and 2 were separated by only 5 days, *e.g.*, too quickly for changing leukocyte subset numbers. Longitudinal comparative proteomics demonstrated that many proteins that are associated with inflammaging ^18^ were attenuated by the rounds of TPE. For instance, pro-inflammatory factors, such as IL-1β, IL-6, and IP-10, stably decreased. In contrast, lipocalin-1 that participates in the sense of taste, and regulators of EGF, IGF, and TGF-β signaling pathways increased, demonstrating a better capacity for tissue maintenance and repair. These finidngs provide important detail on the general observation that TPE reduces the inflammation, which is known to be upregulated by long-haul COVID-19. ^19^ Of note, the smell/taste reception returned to normal in this patient after TPE. In summary, this case study suggests that TPE may alleviate post COVID-19 sequelae *via* positive shifts toward adaptive immunity and tissue repair that are concurrent with reduction of inflammation.

## Methods

The study was approved by the Diagnostics Investigational Review Board ( www.dxirb.com) with study identifier - 7347. The study was performed under the written informed consent (obtained by Dr. Dobri Kiprov) from the patient for the use and publication of the patient’s data. Therapeutic plasma exchange (TPE) was performed as described. ^20^^,^^21^ Briefly, TPE was performed using centrifugal blood cell separator (Spectra Optia, Terumo BCT, Colorado, USA). One plasma volume was removed and replaced with 5% albumin. Each procedure was followed by infusion of 2 gm, 10% Intravenous Gammaglobulin G (IVIG, Octagam 10%, Octapharma, Hoboken, NJ, USA).

### Serum, plasma, and PBMC isolation

Whole blood was collected immediately before (pre) or after (post) TPE procedure in the clinic and processed into serum, plasma and PBMCs (Histopaque density) as described. ^22^

### Real-time polymerase chain reaction (PCR)

Total RNA was extracted from PBMC using the RNeasy mini kit (Qiagen), and the SuperScript III First-Strand Synthesis System (Invitrogen). Real-time PCR was performed on a Bio-Rad iQ5 real-time PCR machine. The primers used for PCR are listed in Table 1.

**Table 1.** Primer sequences for real-time PCR.

Group	Gene name		Sequence
T cells	CD3	Forward	GATGCAGTCGGGCACTCACT
		Reverse	CATTACCATCTTGCCCCCAA
Helper T-cells	CD4	Forward	GCCAACCCAAGTGACTCTGT
		Reverse	TCTCCTGGACCACTCCATTC
Cytotoxic T-cells	CD8	Forward	ACTTGTGGGGTCCTTCTCCT
		Reverse	GTCTCCCGATTTGACCACAG
NK cells	CD94	Forward	GAGCCAGCATTTACTCCAGGAC
		Reverse	GCACAGAGATGCCGACTTTCGT
B cells	B220	Forward	ACA GCC AGC ACC TTT CCT AC
		Reverse	GTG CAG GTA AGG CAG CAG A
	CD19	Forward	AAGGGGCCTAAGTCATTGCT
		Reverse	CAGCAGCCAGTGCCATAGTA
Myeloid cells	CD11b	Forward	CAGCCTTTGACCTTATGTCATGG
		Reverse	CCTGTGCTGTAGTCGCACT
	CD68	Forward	GCTACATGGCGGTGGAGTACAA
		Reverse	ATGATGAGAGGCAGCAAGATGG
IL-2 receptor	CD25	Forward	GAGAAAGACCTCCGCTTCAC
		Reverse	CGAGTGGCTAGAGTTTCCTG
Housekeeping	b-actin	Forward	TGAAGTGTGACGTGGACATC
		Reverse	GGAGGAGCAATGATCTTGAT

### Antibody array

Serum was analyzed on a Ray Biotech human L507 antibody capture array (AAH-BLG-1-4, Raybiotech), processed according to the manufacturer’s protocol. The array slides were imaged by a Molecular Devices 4000b scanner and data were calculated by Genepix. Normalization was done by bult-in positive and negative controls.

### Bioinformatics analysis

The gene ID of differently regulated proteins was performed using DAVID Bioinformatics Resources (version 6.8, https://david.ncifcrf.gov), as well as the GO (Gene Ontology) analysis of the biological process (BP), molecular function (MF) and cellular component (CC), and Kyoto Encyclopedia of Genes and Genomes (KEGG) pathway. Heat maps were performed with ClustVis. ^23^ Volcano plots were constructed with the Graphpad Prism software version 9 (GraphPad software Inc).

### Statistical analysis

All statistical analyses were performed using GraphPad Prism software. All values are expressed as means ± SEM for independent experiments, or SD for replicates. To determine the significance of differences among groups, comparisons were made using Student’s *t*-test. The *P* < 0.05 was considered significant.

## Data availability

### Underlying data

OSF: Underlying data for ‘Therapeutic and immunomodulatory effects of plasmapheresis in long-haul COVID: a case report’ https://doi.org/10.17605/OSF.IO/AXPW7. ^24^

The project contains the following underlying data:

[Dataset_1_qPCR raw data.xlsx] (Raw qPCR data).

[Dataset_2_Antibody array raw data.xlsx] (Antibody array raw data).

[Dataset_3_raw data of diagnosis.pdf] (Diagnosis data).

Data are available under the terms of the Creative Commons Attribution 4.0 International license (CC-BY 4.0).

## Authors’ contributions

DKiprov planned and performed all clinical work, established the IRB approval, provided Figure 1 and the blood samples to the Conboy laboratory and co-wrote the manuscript; DKim provided Figure 2, bio-computation and bioinformatics for Figure 3, and participated in the manuscript writing; ML contributed to Figure 2; CL and EW performed the comparative proteomics that is shown in Figure 3 and CL provided the schematic of Figure 1A; JH, RR and AH provided clinical support; MJK contributed to the planning of this work and co-wrote the manuscript; IC planned, directed, and integrated the study, interpreted the data, and co-wrote the manuscript. All authors agreed with publication of this work.
