# Supplementary material for: Case Report: Therapeutic and immunomodulatory effects of plasmapheresis in long-haul COVID
Source: F1000Res. 2022 Apr 6;10:1189. Originally published 2021 Nov 24. [Version 2] doi: 10.12688/f1000research.74534.2 (PMC9021669; doi:10.12688/f1000research.74534.2)
Supplement: Peer review history

# Peer review: Reviewer response for version 2

**DOI:** 10.5256/f1000research.123022.r129947

**Published:** 2022-04-13

**Authors:** Prashant Nasa

**Affiliations:** 1 Department of Critical Care Medicine, NMC Specialty Hospital, Dubai, United Arab Emirates

Thank you for the revised manuscript. All comments have been satisfactorily answered. I approve the revised version of the manuscript.

Are enough details provided of any physical examination and diagnostic tests, treatment given and outcomes?

Yes

Is the case presented with sufficient detail to be useful for other practitioners?

Yes

Is sufficient discussion included of the importance of the findings and their relevance to future understanding of disease processes, diagnosis or treatment?

Partly

Is the background of the case’s history and progression described in sufficient detail?

Yes

Reviewer Expertise:

Management of COVID-19 related respiratory failure.

I confirm that I have read this submission and believe that I have an appropriate level of expertise to confirm that it is of an acceptable scientific standard.

# Peer review: Reviewer response for version 2

**DOI:** 10.5256/f1000research.123022.r129948

**Published:** 2022-04-07

**Authors:** Bernd Stegmayr

**Affiliations:** 1 Department of Public Health and Clinical Medicine, Umeå University, Umeå, Sweden

Apparently three TPEs were performed. The intervals of TPE are not precisely given by the authors but seem to be 5 days or more? The centrifugal technique, as used here, enables easier removal of large molecules and aggregates (compared to filtration technique) and in such instances would be favorable. Citrate anticoagulation usually used with centrifugation technique also would limit risks for subsequents bleeding if platelets are/get low. Frequent tightly performed TPE with only albumin as replacement may reduce vital plasma compounds very much and give adverse events unless substituted. 

 The present Case report supports knowledge of an important clinical issue. 

 No further comments.

Are enough details provided of any physical examination and diagnostic tests, treatment given and outcomes?

Partly

Is the case presented with sufficient detail to be useful for other practitioners?

Partly

Is sufficient discussion included of the importance of the findings and their relevance to future understanding of disease processes, diagnosis or treatment?

Partly

Is the background of the case’s history and progression described in sufficient detail?

Yes

Reviewer Expertise:

Long term experience with apheresis therapies and research. Specialist Internal Medicine and Nephrology.

I confirm that I have read this submission and believe that I have an appropriate level of expertise to confirm that it is of an acceptable scientific standard.

# Peer review: Reviewer response for version 1

**DOI:** 10.5256/f1000research.78300.r123772

**Published:** 2022-02-25

**Authors:** Prashant Nasa

**Affiliations:** 1 Department of Critical Care Medicine, NMC Specialty Hospital, Dubai, United Arab Emirates

Congratulations authors for the successful outcome of this exploratory study. I have the following points for consideration to make the discussion comprehensive.

1. There is a recent review published in Nature on the pathology of long-haul COVID-19. It will be worth using that in the discussion. ( https://doi.org/10.1038/s41590-021-01104-y). The pathophysiology of long-haul COVID-19 involving imbalance between persistent inflammation on homeostasis should be discussed and provide prospects for future controlled studies.
2. Was plasma removed through therapeutic plasma exchange profiled through comparative proteomics? This would have helped in understanding the degree of reduction of the biomarkers with each session of TPE.
3. Role of TPE and Plasma exchange has shown variable effects and investigators have used them in different combinations. It is worth including them in discussing ( https://www.ijidonline.com/article/S1201-9712(20)32289-X/fulltext)

Are enough details provided of any physical examination and diagnostic tests, treatment given and outcomes?

Yes

Is the case presented with sufficient detail to be useful for other practitioners?

Yes

Is sufficient discussion included of the importance of the findings and their relevance to future understanding of disease processes, diagnosis or treatment?

Partly

Is the background of the case’s history and progression described in sufficient detail?

Yes

Reviewer Expertise:

Management of COVID-19 related respiratory failure.

I confirm that I have read this submission and believe that I have an appropriate level of expertise to confirm that it is of an acceptable scientific standard, however I have significant reservations, as outlined above.

## References

[1] : Pathological sequelae of long-haul COVID. *Nat Immunol* .23(2) : 10.1038/s41590-021-01104-y 194-202 10.1038/s41590-021-01104-y 35105985PMC9127978

[2] : Therapeutic plasma exchange followed by convalescent plasma transfusion in critical COVID-19-An exploratory study. *Int J Infect Dis* .2021;102: 10.1016/j.ijid.2020.10.085 332-334 10.1016/j.ijid.2020.10.085 33157287PMC7607263

# Peer review: Reviewer response for version 1

**DOI:** 10.5256/f1000research.78300.r119963

**Published:** 2022-01-28

**Authors:** Bernd Stegmayr

**Affiliations:** 1 Department of Public Health and Clinical Medicine, Umeå University, Umeå, Sweden

The Case Report using Plasmapheresis (apheresis) for long-haul Covid increases awareness of the complexity of the disease and the possibilities to immunomodulate by apheresis. 

 The topic is very important and the concept well described. The case is also well described. 

 There is a lack of information that should be added:

1. What type of apheresis procedure (centrifuge or filtration, leukapheresis?) was used?
2. What type and doses of anticoagulation during apheresis was used? (citrate, LMWH, heparin?)
3. Was a maintenance pharmacological therapy used also after initiation of apheresis to prohibit antibodies to increase?
4. If so, what were the pharmacological doses/kg body weight and/day of such medication (or interval)?
5. What were the time intervals between the apheresis procedures?
6. Did you try to investigate for specific antibodies?
7. You could add the concept of apheresis for antibody removal such as described for VITT by Rock et al. 2021 ^1^

Are enough details provided of any physical examination and diagnostic tests, treatment given and outcomes?

Partly

Is the case presented with sufficient detail to be useful for other practitioners?

Partly

Is sufficient discussion included of the importance of the findings and their relevance to future understanding of disease processes, diagnosis or treatment?

Partly

Is the background of the case’s history and progression described in sufficient detail?

Yes

Reviewer Expertise:

Long term experience with apheresis therapies and research. Specialist Internal Medicine and Nephrology.

I confirm that I have read this submission and believe that I have an appropriate level of expertise to confirm that it is of an acceptable scientific standard, however I have significant reservations, as outlined above.

## References

[1] : Therapeutic plasma exchange (TPE) as a plausible rescue therapy in severe vaccine-induced immune thrombotic thrombocytopenia. *Transfus Apher Sci* .2021;60(4) : 10.1016/j.transci.2021.103174 103174 10.1016/j.transci.2021.103174 34088601
